# Recurrent febrile seizures and serum cytokines: a controlled follow-up study

**DOI:** 10.1038/s41390-022-02282-7

**Published:** 2022-09-23

**Authors:** Maria K. Hautala, Heli M. Helander, Tytti M-L Pokka, Ulla V. Koskela, Heikki M. J. Rantala, Matti K. Uhari, Timo J. Korkiamäki, Virpi Glumoff, Kirsi H. Mikkonen

**Affiliations:** 1 PEDEGO Research Unit – Research Unit for Pediatrics, Pediatric Neurology, Pediatric Surgery, Child Psychiatry, Dermatology, Clinical Genetics, Obstetrics and Gynecology, Otorhinolaryngology and Ophthalmology, Medical Research Center Oulu (MRC Oulu), University of Oulu, Oulu, Finland; 2 Department of Pediatrics and Adolescent Medicine, Oulu University Hospital, Oulu, Finland; 3 Research Unit of Biomedicine, University of Oulu, Oulu, Finland; 4 Epilepsia Helsinki, Division of Child Neurology, Children’s Hospital, and Pediatric Research Center, Helsinki University Hospital and University of Helsinki, Helsinki, Finland

## Abstract

**Background:**

The role of cytokines in the pathogenesis of febrile seizures (FSs) is unclear, and information regarding cytokine production outside of FS episodes is scarce.

**Methods:**

In our controlled follow-up study of patients with FSs, we compared the levels of 12 serum cytokines after the patients’ first FSs, during febrile episodes without FSs, after recurrent FSs, during healthy periods after FSs, and between patients and controls.

**Results:**

Two-hundred fifty-one patients with first FS participated in the study, of whom 17 (mean age 1.6 years, SD 0.7) with recurrent FSs completed the protocol as required by the sample size calculations. The mean IL-1RA level was higher after the first FSs (2580 pg/mL, SD 1516) than during febrile episodes without FSs (1336 pg/mL, SD 1364, *P* = 0.006) and healthy periods after FSs (474 pg/mL, SD 901, *P* = 0.001). IL-1RA levels were also higher during first (2580 pg/mL) and recurrent FSs (2666 pg/mL, SD 1747) in comparison with febrile controls (746 pg/mL, SD 551) (*P* < 0.001 and *P* = 0.001, respectively), but there was no difference in the IL-1RA between febrile episodes without FSs and febrile controls.

**Conclusions:**

Patients with FSs produce stronger inflammatory reactions during febrile episodes with FSs compared with febrile episodes without FSs and febrile controls.

**Impact:**

- In patients with FSs, IL-1RA was higher following first FS than during febrile episodes without FSs and healthy periods after FSs.
- IL-1RA was higher in patients with FSs following first and recurrent FSs than in febrile controls.
- There was no significant difference in IL-1RA between febrile episodes of patients without FSs and febrile controls.
- Using IL-1RA as a surrogate marker of IL-1 axis activity, our results indicate that patients with FSs produced stronger inflammatory reactions during FS episodes but not during other febrile episodes or healthy periods after FSs.
- Cytokines may play a role in pathogenesis of FSs.

## Introduction

Febrile seizures (FSs) occur in 2–5% of children under 6 years of age and recur in 20–30% of the patients in subsequent febrile episodes.^1^ FSs cause significant emotional and economic burdens. The exact pathogenesis of FSs is unknown, but during a specific developmental stage, certain genetically determined features of the central nervous and inflammatory systems may induce responses that cause seizures during febrile infections. This notion is supported by the recent identification of new genetic loci that are functionally related to fever responses and neuronal excitability.^2^

Prostaglandins and cytokines mediate a host’s inflammatory response to an infection. It has been suggested that virus-induced release of cytokines in the central nervous system is one of the factors that causes neuronal hyperexcitability, which can lead to seizures.^3,4^ In animal studies, various cytokines have been found to either enhance or suppress provoked convulsions.^5,6^ Skotte et al.^2^ found an association between FSs and single nucleotide polymorphisms in the anti-inflammatory cytokine interleukin (IL)-10 genes. Altered levels of the pro-inflammatory cytokines IL-1β, IL-6, tumor necrosis factor (TNF)-α and the anti-inflammatory cytokines IL-1RA and IL-10 have been found in sera and cerebrospinal fluid from patients with FSs immediately following seizures,^7–10^ but information regarding cytokine levels in patients with FSs outside of febrile episodes with FSs is scarce.

Previously, we showed that antipyretics were ineffective in lowering temperatures during infections that led to seizures.^11^ Considering this persistent fever response, we hypothesized that patients’ cytokine levels differed between febrile infections without seizures and infections that led to FS. In addition, we hypothesized that cytokine levels differed in patients with FSs compared to other febrile children and healthy adults without FSs.

## Methods

### Study population and study design

This was a controlled follow-up study. The Ethics Committee (Institutional Review Board) of Northern Ostrobothnia Hospital District, Finland, found the study plan acceptable (diary number 6/2011). Informed consent was obtained from the guardians of the participating children and from the adult study participants. The study was conducted from January 2012 to May 2019 in Oulu University Hospital, Finland. This hospital is the only hospital in the area with a pediatric emergency room (ER) and pediatric wards. The hospital’s primary catchment area has a population of 85,000 children aged <16 years, and the secondary catchment area has a population of 148,000 in the same age group. The study cohort was most likely representative of typical FSs among the population, as the current Finnish guidelines recommend that all patients having their first FS should be referred to a pediatric ER.

**Fig. 1:**
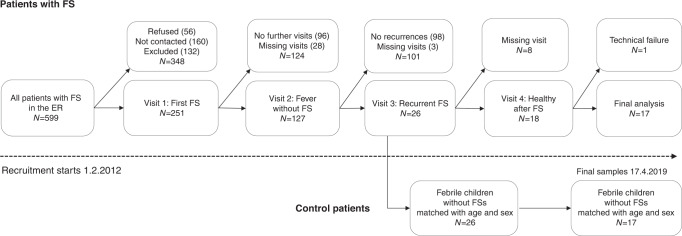
Study profile Flow chart of the recruitment and follow-up visits of patients with FSs and controls. ER emergency room, FS febrile seizure.

All patients visiting the hospital’s pediatric ER with their first FS were invited to join the study. We chose to exclude patients younger than 6 months and older than 6 years. The exclusion criteria for patients with FSs were a history of previous afebrile seizures or epilepsy, any syndrome or cerebral malformation that markedly increased the risk of epilepsy, an evident central nervous system infection, a significant electrolyte imbalance, a body temperature <38 °C, and psoriasis. We only included patients with at least one native Finnish-speaking parent to avoid communication problems. The study clinician/nurse contacted the family of each patient after the first seizure. The study protocol included four visits for each patient with FSs: one for the first FS, one for a febrile infection without FSs, one for a recurrent FS, and one while the patient was healthy after a seizure (Fig. 1). The order of the revisits varied because some patients had several FS episodes before having a febrile episode without FSs. Samples taken during healthy periods after seizures were programmed to be collected 3 weeks after the recurrent FS; however, in some cases, they were postponed due to recurrent infections.

We selected one febrile control patient for each patient with FSs after the index patient had had a recurrent FS. We recruited the controls from hospital patients with febrile infections and body temperatures >38 °C. These controls were matched by age (±6 months) and sex. We also selected unrelated healthy adult control patients, matched by sex. The exclusion criteria for the controls were a history of FS, epilepsy, any syndrome or cerebral malformation that markedly increased the risk of epilepsy, an evident central nervous system infection, and psoriasis. Exclusion of patients with psoriasis was needed for another part of the study. No patients were excluded because of this criterion.

**Fig. 2:**
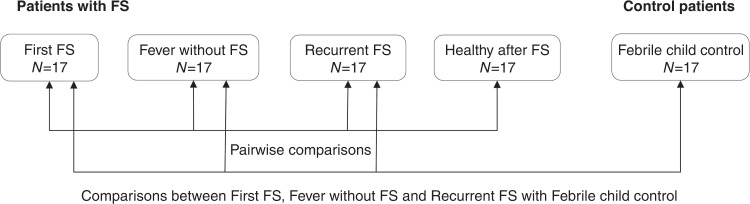
Diagram of the statistical comparisons of cytokine levels between study groups. FS febrile seizure.

By comparing patients with FSs across different time points and to febrile children without FSs, we aimed to determine whether the cytokine production in patients with FSs varied in different situations and whether there was a fundamental difference in cytokine responses between these two groups (Fig. 2). We specifically chose to use healthy adults as controls in addition to the febrile children. It is unethical to draw blood samples from healthy infants only for study purposes. In addition, the cytokine levels that are measured from clinical samples vary among different laboratories. As there are more available data regarding the baseline cytokine values in healthy adults, we wanted to give the reader of the article the possibility to compare our values to the values of their own. Thus, we had to compare the cytokine levels of patients with FSs during healthy periods with those of healthy adults.

For c-reactive protein analysis and cytokine measurement purposes, we collected a blood sample from a peripheral vessel from each patient at each study visit. The blood samples were centrifugated, and the sera were stored at –80 °C until the analysis. For the FS-associated visits, we obtained the samples within 24 h post-seizure. A nasopharyngeal respiratory virus sample was taken during each febrile study visit for multiplex polymerase chain reaction assay purposes. Each of these samples was either a nasopharyngeal aspirate or swab.

Patient data were systematically collected from the medical records and through interviews with the parents using a structured questionnaire. For each patient, these data included demographic features; family history of FSs; clinical diagnosis; symptoms of the infection; starting date and time of the infection and the fever; primary focus of the infection; date, time, type, and duration of FS; body temperature on arrival to the ER; highest body temperature during the period measured by the parents, paramedics, or hospital staff; and the use of antipyretics or antiepileptic emergency medications. Complex FSs were defined as focal seizures, seizures lasting more than 15 min, or recurrent seizures during a 24-h period.

### Cytokine measurement

The concentrations of pro-inflammatory cytokines (IL-1β, IL-2, IL-5, IL-6, IL-8, IL-12p70, TNF-α, TNF-β, and IFN-γ) and anti-inflammatory cytokines (IL-4, IL-10, and IL-1RA) were measured using a commercially available bead-based FlowCytomix™ Multiple Analyte Detection System according to the manufacturer’s instructions (eBioscience, Thermo Fisher Scientific, Waltham, Massachusetts). The detection was carried out with a BD LSRFortessa™ Flow Cytometer (BD Biosciences, San Jose, California). For IL-8, the lower limit of quantification (LLQ) was 13.72 pg/mL; for the rest of the cytokines, the LLQ was 27.43 pg/mL. For statistical purposes, we expressed serum cytokine levels below the LLQ as the midpoint between zero and the LLQ of the analyte. If the program gave a specific value below the LLQ, we left it unchanged.

Ideally, cytokine measurements are performed immediately after samples are taken. However, to execute our follow-up protocol, we were required to choose between a long storage time or several cytokine analyses performed at different time points. We concluded that allowing a longer storage time would cause less bias because the storage times of samples from patients and controls would be similar and, thus, comparable. The samples were stored at –80 °C to slow the degradation process.

### Outcomes

We compared the cytokine levels of patients with FSs in four different situations: within 24 h after the first FS, during febrile infection without FSs, within 24 h after a recurrent FS, and while a patient was healthy after FS. This way, patients with FS served as controls for themselves. We also compared the cytokine levels of patients with FSs with those of two control populations: febrile control children without FSs (matched by age and sex) and healthy adult controls (matched by sex).

### Statistical analyses

We calculated the sample size using an α error of 5% and a *β* error of 20%. We chose IL-1β as the primary cytokine based on its central role in the formation of febrile response, the results of animal studies regarding provoked convulsions, and the results of genetic and clinical studies examining cytokines in FSs.^5,8,12^ We selected the 25 pg/mL standard deviation (SD) of IL-1β concentration from an experimental study that evaluated the effect of priming macrophages with interferon-γ on the cytokine production of the cells.^13^ We estimated that this change of one SD could be regarded as the smallest clinically significant change. To our knowledge, earlier studies regarding cytokine production in patients with FSs have not used sample size calculations, and there are no clinical studies defining the smallest clinically significant change in IL-1β concentration. Our estimates resulted in a sample size of 16 patients for each group.

The background characteristics of the patients with FSs and control patients were tested a priori using the Friedman test, which is a generally acknowledged way to measure differences between repeated measurements among multiple groups. If the Friedman test gave a statistically significant *P*-value, we used the Wilcoxon-signed rank test as a post-hoc test for continuous variables. For dichotomous variables, we used a sign test. The cytokine levels were expressed as means and SDs. Differences in serum cytokine levels between visits of patients with FSs and between patients with FSs and controls were compared a priori using the Friedman test. If the Friedman test gave a significant *P*-value, a post-hoc Wilcoxon-signed rank test was used. Cytokine levels were given as medians and interquartile ranges (IQRs). All analyses were performed using IBM Statistics for Windows version 27 (IBM Corp., Armonk, New York) and StatsDirect statistical software version 3 (StatsDirect Ltd., Birkenhead, UK). Figures were drawn using GraphPad Prism version 9 (Graphpad, San Diego, California) and SmartDraw version 2008 (SmartDraw Software, LLC, The Woodlands, Texas).

## Results

### Patient characteristics

In total, 599 patients with FSs were treated at the pediatric ER during the study period. Of them, 307 patients were invited to join the study; 251 patients accepted. Of the 251 patients with FSs, 60 (24%) had recurrent seizures. Altogether, 18 patients with recurrent seizures completed the study protocol; however, the cytokine analysis failed for 1 patient. Therefore, 17 patients were included in the final analysis (Fig. 1).

Overall, 10 (59%) of the 17 patients with FSs were boys (Table 1). The mean age of the patients at the first FS episode was 1.6 years (SD 0.7). The mean duration of the first FS was 5 min and 45 s (median 3:00 min, range 01:00 to 25:00 min). The mean duration of recurrent FSs was 5 min and 57 s (median 3:00 min, range 0:30 to 20:00 min). Overall, 8 (47%) patients with recurrent FSs had complex FSs (Table 1).

**Table 1:** Background characteristics of patients with FSs during the first FSs, during febrile episodes without FSs, during recurrent FSs, and during a healthy period after FSs and of controls.

	Patients with FSs				Controls		*P*-values		
Background characteristics	First FS *N* = 17	Fever w/o FS *N* = 17	Recurrent FS *N* = 17	Healthy after FS *N* = 17	Febrile children *N* = 17	Healthy adults *N* = 17	First FS vs. Febrile children	Fever w/o FS vs. Febrile children	Recurrent FS vs. Febrile children
Male, *n* (%)	10 (58.8)	10 (58.8)	10 (58.8)	10 (58.8)	10 (58.8)	10 (58.8)			
Age, years, mean (SD)	1.6 (0.7)	2.4 (1.4)	2.5 (1.4)	2.8 (1.5)	2.2 (1.3)	32.9 (6.7)			
CRP, mg/L (SD)	12 (13)	29 (36)	24 ((29)	1 (2)	85 (70)	0 (1)	0.001*	0.017*	0.009*
BT on arrival, °C (SD)	38.5 (0.7)	38.2 (0.8)	38.3 (0.7)	-	38.4 (1.0)	-	0.877	0.712	0.679
Antipyretic medication, *n* (%)	17 (100)	8 (47)	14 (82)	0 (0)	15 (88)	0 (0)	0.500	>0.999	0.500
Timing of the cytokine sample after fever rise, h (SD)	11 (13)	24 (25)	11 (8)	-	125 (177)	-	0.003*	0.046*	0.012*
Chronic condition									
Allergy or atopy, *n* (%)	2 (11.8)	2 (11.8)	2 (11.8)	2 (11.8)	3 (17.6)	0 (0)			
Asthma, *n* (%)	1 (5.9)	1 (5.9)	0 (0)	0 (0)	3 (17.6)	0 (0)			
Congenital malformation^a^, *n* (%)	1 (5.9)	1 (5.9)	1 (5.9)	1 (5.9)	0 (0)	0 (0)			
Other^b^, *n* (%)	0 (0)	0 (0)	0 (0)	0 (0)	1 (5.9)	2 (11.8)			
Diagnosis on arrival									
Respiratory infection, *n* (%)	15 (88.2)	13 (76.5)	12 (70.6)	-	4 (23.5)	-			
Gastroenteritis, *n* (%)	0 (0)	0 (0)	0 (0)	-	1 (5.9)	-			
Other viral infection, *n* (%)	1 (5.9)	2 (11.8)	5 (29.4)	-	3 (17.6)	-			
Other reaction^c^, *n* (%)	0 (0)	2 (11.8)	0 (0)	-	1 (5.9)	-			
Severe bacterial infection, *n* (%)	1 (5.9)	0 (0)	0 (0)	-	8 (47.1)	-			
Duration of FS, min, mean (median, range)	5.75 (3, 1–25)	-	5.95 (3, 0.5–20)	-	-	-			
Complex FSs, *n* (%)	8 (47.1)	-	8 (47.1)	-	-	-			

*FS* febrile seizure, *CRP* c-reactive protein, *BT* body temperature, w/o without.

^a^Congenital malformation other than CNS malformation: Congenital fibular hemimelia.

^b^Other chronic conditions: hypothyreosis, familial hypercholesterolemia.

^c^Other reaction: febrile reaction conjugated to a vaccination, Kawasaki disease.

^*^Statistically significant *P* < 0.05.

### Cytokine concentrations in patients with FSs

#### Anti-inflammatory cytokines

**Fig. 3:**
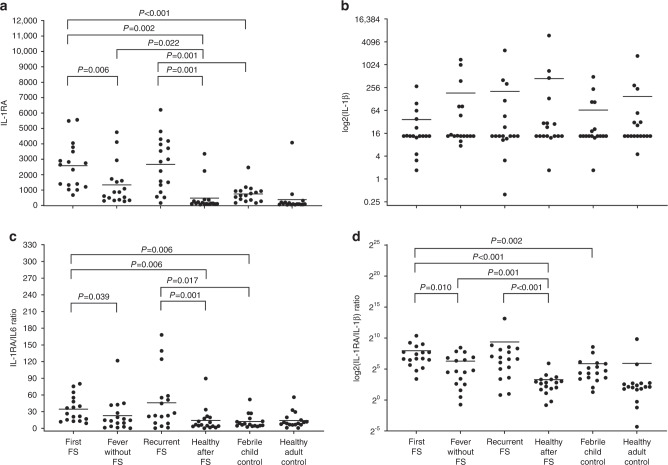
Graph of the comparisons of the cytokine levels between study groups. Serum (**a**) IL-1RA and (**b**) IL-1β levels, and (**c**) IL-1RA/IL-6 and (**d**) IL-1RA/IL-1β ratios in patients with FSs during the first FS, during febrile episodes without FSs, during recurrent FSs and during a healthy period after FSs and in controls. For IL-1β and the IL-1RA/IL-1β ratio, the *y*-axis illustrated in logarithmic scale. FS febrile seizure.

The IL-1RA levels measured after first FSs (mean 2580 pg/mL [SD 1516]; median 2640 [IQR 1238–3713]) were significantly higher than those measured during febrile episodes without FS (mean 1336 pg/mL [SD 1364]; median 867 [IQR 344–1674]; *P* = 0.006) and those measured during healthy periods after FSs (mean 474 pg/mL [SD 901]; median 134 [IQR 97–345]; *P* = 0.002; Table 2, Fig. 3). The IL-1RA levels were somewhat higher after recurrent FSs (mean 2666 pg/mL [SD 1747]; median 2843 [IQR 1375–4130]) than they were during febrile episodes without FSs (mean 1336 pg/mL); however, this difference was not statistically significant (*P* = 0.124). The IL-1RA levels were also significantly higher during febrile episodes without FSs (mean 1336 pg/mL) than they were during healthy periods after FSs (mean 474 pg/mL). There was no difference in the IL-1RA levels between the first FSs (mean 2580 pg/mL) and recurrent FSs (mean 2666 pg/mL; Table 2, Fig. 3). The levels of IL-10 were significantly higher during healthy periods after FSs than they were after first FS episodes (mean 1317 pg/mL [SD 5184] vs. 66 pg/mL [SD 86]; *P* = 0.0039; Table 2).

**Table 2:** Cytokine concentrations (pg/mL) expressed as means (SD) for patients with FSs during the first FSs, during febrile episodes without FSs, during recurrent FSs, and during a healthy period after FSs.

	First FS *N* = 17	Fever w/o FS *N* = 17	Recurrent FS *N* = 17	Healthy after FS *N* = 17	*P*-values			
Cytokine					First FS vs. Fever w/o FS	First FS vs. Healthy after FS	Fever vs. Healthy after FS	Recurrent FS vs. Healthy after FS
Anti-inflammatory								
IL-1RA (SD)	2580 (1516)	1336 (1364)	2666 (1747)	474 (901)	0.006*	0.002*	0.022*	0.001*
IL-4 (SD)	90 (169)	217 (418)	272 (791)	693 (2258)	NS	NS	NS	NS
IL-10 (SD)	66 (86)	137 (311)	366 (1231)	1317 (5184)	NS	0.039*	NS	NS
Pro-inflammatory								
IL-1β (SD)	37 (66)	184 (398)	203 (584)	442 (1447)	NS	NS	NS	NS
IL-2 (SD)	151 (286)	345 (621)	529 (1468)	1102 (3554)	NS	NS	NS	NS
IL-5 (SD)	189 (372)	504 (1135)	4042 (15,721)	9835 (39,587)	NS	NS	NS	NS
IL-6 (SD)	114 (98)	350 (1010)	933 (3308)	1630 (6181)	NS	NS	0.005*	NS
IL-8 (SD)	1245 (2852)	362 (900)	926 (1602)	833 (1429)	NS	NS	NS	NS
IL-12p70 (SD)	16 (6)	61 (143)	36 (72)	157 (546)	NS	NS	NS	NS
TNF-α (SD)	26 (37)	194 (519)	137 (414)	903 (3427)	0.05*	0.037*	NS	NS
TNF-β (SD)	54 (127)	183 (377)	287 (788)	608 (2034)	NS	NS	NS	NS
IFN-γ (SD)	306 (726)	1210 (2718)	3528 (13,293)	14058 (55,267)	NS	NS	NS	NS
Ratios								
IL-1RA/IL-1β (SD)	250 (325)	78 (102)	665 (2168)	10 (14)	0.01*	<0.001*	0.001*	<0.001*
IL-1RA/IL-6 (SD)	35 (23)	23 (29)	46 (51)	14(21)	0.039*	0.006*	NS	0.001*
IL-1RA/IL-8 (SD)	60 (88)	100 (217)	29 (59)	4 (7)	NS	<0.001*	0.011*	0.004*

*FS* febrile seizure, *IL* interleukin, *IFN* interferon, *IL-1RA* interleukin 1-receptor antagonist, *NS* not significant, *TNF* tumor necrosis factor, *w/o* without.

^*^Statistically significant *P* < 0.05, Wilcoxon-signed rank test.

#### Pro-inflammatory cytokines

The IL-6 levels were significantly higher during healthy periods after FSs than they were during febrile episodes without FSs, with mean values of 1630 pg/mL (SD 6181) and 350 pg/mL (SD 1010), respectively (*P* = 0.005). TNF-α levels were significantly higher during febrile episodes without FSs than after the first FS episodes (mean 194 pg/mL [SD 519] vs. 26 pg/mL [SD 37]; *P* = 0.05) and during healthy periods after FSs compared to after first FS episodes (mean 903 pg/mL [SD 3427] vs. 26 pg/mL [SD 37]; *P* = 0.037; Table 2).

#### Ratios of anti-inflammatory cytokines to pro-inflammatory cytokines

The mean IL-1RA:IL-1β ratio was higher after the first FSs than during febrile episodes without FSs (250 [SD 325] vs. 78 [SD 102]; *P* = 0.01; Table 2, Fig. 3). The mean IL-1RA:IL-1β ratio was also somewhat higher after recurrent FS episodes than during febrile episodes without FSs (665 [SD 2168] vs. 78 [SD 102]), but this difference was not statistically significant (*P* = 0.149). There was no significant difference in the IL-1RA:IL-6 ratio between the first and recurrent FS episodes. The mean IL-1RA:IL-6 ratio was significantly higher after the first FS episodes (35 [SD 23]) than during febrile episodes without FSs (23 [SD 29]; *P* = 0.039; Table 2, Fig. 3).

### Comparisons of cytokine concentrations between patients with FSs and controls

#### Anti-inflammatory cytokines

The IL-1RA levels were significantly higher following both first FSs (mean 2580 pg/mL [SD 1516]; median 2640 [IQR 1238–3713]) and recurrent FSs (mean 2666 pg/mL [SD 1747]; median 2843 [IQR 1375–4130]) compared with the levels in febrile control children (mean 746 pg/mL [SD 551]; median 652 [IQR 368–946]; *P* < 0.001 and *P* = 0.001, respectively). The IL-1RA levels of patients with FS during febrile episodes without FSs (mean 1336 pg/mL [SD 1364]; median 867 [IQR 345–1674]) did not differ from those of febrile control children (mean 746 pg/mL; Table 3, Fig. 3). There were no significant differences between the patients with FSs and febrile control children with regard to any other anti-inflammatory cytokines (Table 3).

**Table 3:** Cytokine concentrations (pg/mL) expressed as means (SDs) in patients with FSs during first FSs, during febrile episodes without FSs, during recurrent FSs, and during a healthy period after FS and in controls.

	FS patients				Controls		*P*-values		
Cytokine	First FS *N* = 17	Fever w/o FS *N* = 17	Recurrent FS *N* = 17	Healthy after FS *N* = 17	Febrile children *N* = 17	Healthy adults *N* = 17	First FS vs. Febrile children	Recurrent FS vs. Febrile children	Healthy after FS vs. Healthy adults
Anti-inflammatory									
IL-1RA (SD)	2580 (1516)	1336 (1364)	2666 (1747)	474 (901)	746 (551)	382 (968)	<0.001*	0.001*	NS
IL-4 (SD)	90(169)	217 (418)	272 (791)	693 (2258)	78 (136)	64 (76)	NS	NS	NS
IL-10 (SD)	66 (86)	137 (311)	366 (1231)	1317 (5184)	76 (140)	47 (87)	NS	NS	NS
Pro-inflammatory									
IL-1β (SD)	37 (66)	184 (398)	203 (584)	442 (1447)	65 (125)	149 (417)	NS	NS	NS
IL-2 (SD)	151 (286)	345 (621)	529 (1468)	1102 (3554)	495 (1106)	182 (312)	NS	NS	NS
IL-5 (SD)	189 (372)	504 (1135)	4042 (15,721)	9835 (39,587)	1139 (3894)	358 (905)	NS	NS	NS
IL-6 (SD)	114 (98)	350 (1010)	933 (3308)	1630 (6181)	110 (116)	42 (67)	NS	NS	0.034*
IL-8 (SD)	1245 (2852)	362 (900)	926 (1602)	833 (1429)	325 (372)	267 (578)	NS	NS	NS
IL-12p70 (SD)	16 (6)	61 (143)	36 (72)	157 (546)	20 (26)	16 (9)	NS	NS	NS
TNF-α (SD)	26 (37)	194 (519)	137 (414)	903 (3427)	51 (111)	21 (22)	NS	NS	NS
TNF-β (SD)	54 (127)	183 (377)	287 (788)	608 (2034)	56 (100)	69 (138)	NS	NS	NS
IFN-γ (SD)	306 (726)	1210 (2718)	3528 (13,293)	14058 (55,267)	218 (383)	104 (206)	NS	NS	NS
Ratios									
IL-1RA/IL-1β (SD)	250 (325)	78 (102)	665 (2168)	10 (14)	58 (96)	60 (219)	0.002*	NS	NS
IL-1RA/IL-6 (SD)	35 (23)	23 (29)	46 (51)	14 (21)	12 (13)	14 (14)	0.006*	0.017*	NS
IL-1RA/IL-8 (SD)	60 (88)	100 (217)	29 (59)	4 (7)	20 (35)	21 (37)	NS	NS	NS

*FS* febrile seizure, *IL* interleukin, *IFN* interferon, *IL-1RA* interleukin 1-receptor antagonist, *NS* not significant, *TNF* tumor necrosis factor, *w/o* without.

^*^Statistically significant *P* < 0.05, Wilcoxon-signed rank test.

#### Pro-inflammatory cytokines

Regarding pro-inflammatory cytokines, only the levels of IL-6 were significantly higher in patients with FSs during healthy periods after FSs (110 pg/mL [SD 116]) than in healthy adults (42 pg/mL [SD 67]; *P* = 0.034; Table 3).

#### Ratios of anti-inflammatory cytokines to pro-inflammatory cytokines

The mean IL-1RA:IL-1β ratio was significantly higher in the first FS episodes of patients with FSs (250 [SD 325]) than in febrile control children (58 [SD 96]; *P* = 0.002; Table 3, Fig. 3). The same was true for the mean IL-1RA:IL-6 ratio (35 [SD 23] and 12 [SD 13], respectively; *P* = 0.006). The mean IL-1RA:IL-6 ratio was also significantly higher after recurrent FSs of patients with FSs (46 [SD 51]) than in febrile control children (12 [SD 13]; *P* = 0.017; Table 3, Fig. 3).

## Discussion

Our controlled follow-up study compared the cytokine levels in patients with FSs across different situations and to matched controls. We found that in patients with FSs, the levels of IL-1RA were significantly higher immediately following FSs than during febrile episodes without FSs and during healthy periods after FSs. In addition, IL-1RA levels were higher in patients with FS during first and recurrent FS episodes than in febrile control children, but there was no significant difference in IL-1RA levels between febrile episodes without FSs and control patients. We also found similar differences in the IL-1RA:IL-1β and IL-1RA:IL-6 ratios between these groups. Our results indicate that patients with FSs produce exaggerated inflammatory responses during FS episodes but not during other febrile episodes or during healthy periods after FSs.

It has been suggested that IL-1RA is one of the best surrogate markers of IL-1β activity, which, in turn, has been shown to cause seizures.^3,4,14,15^ IL-1RA acts by limiting IL-1β-mediated pro-inflammatory actions, and clinical studies have shown that IL-1RA is robustly released following IL-1β induction activity. However, the measurement of IL-1β itself may not be an ideal marker of IL-1 system activation, because very small concentrations of IL-1β are required to initiate robust inflammatory responses. In addition, IL-1β binds to large proteins, which may reduce its detection in clinical samples by 50%.^14^

Our results suggest that patients with FSs produce higher levels of cytokines only during infections that lead to FSs. These results confirm the findings from our previous randomized controlled trial, in which we found that the maximum body temperature measured during a febrile episode was higher in a FS episode than in a febrile episode without FSs. Furthermore, in the same study, all antipyretics studied were ineffective in lowering body temperature during FS episodes, though they were effective in febrile episodes without FSs for the same patients.^11^ In another recent study, we discovered that patients with FSs produced stronger febrile reactions to respiratory viruses than controls matched with the same respiratory virus.^16^ Such a febrile reaction may reflect the cytokine reaction of the host. These exaggerated reactions seem to be influenced by several factors; it may be that different viruses cause distinct cytokine reactions. In the current study, we were unable to match controls for the same respiratory virus; therefore, we could not evaluate the effects of different viruses on cytokine production. However, we aimed to ensure the viral etiology to be as equal as possible by recruiting the patients and the matched controls as simultaneously as possible.

Presuming that IL-1RA can be regarded as a surrogate marker of IL-1 axis activity, we demonstrated that patients with FSs produced higher inflammatory responses than febrile control patients without FSs. Furthermore, as there were no significant differences in the cytokine levels between patients with FSs during febrile episodes without FS and febrile control patients, we were able to show that the altered cytokine levels were actually linked to the seizures. This raises the question whether the increase in IL-1RA is a result of the fever and the seizure itself or whether this increase precedes the seizure. As pointed out by a recent, large, genome-wide association study of FSs, there seem to be several factors affecting the unique febrile response of patients with FSs.^2^ Once these factors are confirmed and the functions of the specific cytokines are thoroughly understood, it will be possible to develop targeted medications to resolve these febrile responses and prevent FSs. However, before this can become possible, there is a need for both functional and genetic studies on this subject.

Rather than a single, specific cytokine, the balance between pro- and anti-inflammatory cytokines might be essential in the multifactorial pathogenesis of FSs. We found significant differences in the ratios of the anti-inflammatory IL-1RA and the pro-inflammatory IL-1β and IL-6 when comparing patients’ first FS episodes with their febrile episodes without FSs. There were also significant differences between first FS episodes and febrile control patients. These results are consistent with previous findings.^8^ The levels of cytokines are highly co-dependent, so reporting isolated values can be misleading, and we recommend that future studies assess cytokine activity as a whole—for example, with network analysis. Mathematical network analysis is a computer-based method of modeling the relationships between several co-dependent factors; therefore, it is suitable for studying the function of cytokine networks.

Cytokines are highly unstable substances, and the timing of sample collection is complicated because cytokines have short half-lives and peak at different points of time. The technical difficulty of measuring cytokines may, in part, explain the contradictions between the results of the current study and those of earlier studies of cytokine levels in patients with FSs.^7–10,17–21^ In the present study, IL-1RA and IL-6 were the only cytokines that demonstrated significant differences between study patients and control patients. The level of IL-1RA, which is described as a reliable indicator of IL-1 axis activity, rises several hours after pro-inflammatory cytokines, reaching its peak about 24 h after a seizure; therefore, we chose a sampling time limit of 24 h after a seizure. This time limit allowed us to standardize the sampling times between febrile cases and controls. Furthermore, it would have been impossible to acquire samples from control patients immediately after the rise of fever. In general, the cytokine levels were likely lowered by the degradation caused by the long storage time. This may mean that the differences in the cytokine levels between groups are actually larger than what we found.

The strengths of the present study include the use of sample size calculations and the versatile study setting that included follow-ups of patients with FSs as well as comparisons between patients’ multiple visits and with matched controls. A limitation of the study is that the cytokine levels may have been affected by the long storage time due to the long time the study lasted. Also, we did not have healthy children as controls.

## Conclusions

According to our results, the levels of IL-1RA were significantly higher in patients with FSs following their first FS episodes than during febrile episodes without FSs and healthy periods after FSs in the same patients. In addition, IL-1RA levels were higher in patients with FSs during first and recurrent FS episodes than in febrile control children, but there was no significant difference in the IL-1RA level between the patients’ febrile episodes without FSs and the febrile control patients. Our results indicate that patients with FSs produce exaggerated inflammatory reactions during FS episodes but not during other febrile episodes or healthy periods after FSs.

## Data Availability

The datasets generated during and/or analyzed during the current study are available from the corresponding author on reasonable request.
